# Genetic Confirmation and Identification of Novel Variants for Glanzmann Thrombasthenia and Other Inherited Platelet Function Disorders: A Study by the Korean Pediatric Hematology Oncology Group (KPHOG)

**DOI:** 10.3390/genes12050693

**Published:** 2021-05-06

**Authors:** Eu Jeen Yang, Ye Jee Shim, Heung Sik Kim, Young Tak Lim, Ho Joon Im, Kyung-Nam Koh, Hyery Kim, Jin Kyung Suh, Eun Sil Park, Na Hee Lee, Young Bae Choi, Jeong Ok Hah, Jae Min Lee, Jung Woo Han, Jae Hee Lee, Young-Ho Lee, Hye Lim Jung, Jung-Sook Ha, Chang-Seok Ki, on behalf of the Benign Hematology Committee of the Korean Pediatric Hematology Oncology Group (KPHOG)

**Affiliations:** 1 Department of Pediatrics, Pusan National University School of Medicine, Pusan National University Children’s Hospital, Yangsan 50612, Korea; 41sirius@hanmail.net (E.J.Y.); limyt@pusan.ac.kr (Y.T.L.); 2 Department of Pediatrics, Keimyung University School of Medicine, Keimyung University Dongsan Hospital, Daegu 42601, Korea; 3 Department of Pediatrics, Keimyung University School of Medicine, Keimyung University Daegu Dongsan Hospital, Daegu 41931, Korea; kimhs@dsmc.or.kr; 4 Department of Pediatrics, University of Ulsan College of Medicine, Asan Medical Center Children’s Hospital, Seoul 05505, Korea; hojim@amc.seoul.kr (H.J.I.); pedkkn@gmail.com (K.-N.K.); taban@hanmail.net (H.K.); 5 Department of Pediatrics, Korea Cancer Center Hospital, Seoul 01812, Korea; mint7803@nate.com; 6 Department of Pediatrics, Gyeongsang National University College of Medicine, Gyeongsang National University Hospital, Jinju 52727, Korea; espark@gnu.ac.kr; 7 Department of Pediatrics, Cha Bundang Medical Center, Cha University, Seongnam 13496, Korea; nangs@hanmail.net; 8 Department of Pediatrics, Ajou University School of Medicine, Ajou University Hospital, Suwon 16499, Korea; zero-ship@hanmail.net; 9 Department of Pediatrics, Daegu Fatima Hospital, Daegu 41199, Korea; johah@med.yu.ac.kr; 10 Department of Pediatrics, Yeungnam University College of Medicine, Daegu 42415, Korea; mopic@hanmail.net; 11 Department of Pediatrics, Yonsei University College of Medicine, Yonsei University Health System, Seoul 03722, Korea; jwhan@yuhs.ac; 12 Department of Pediatrics, Chungbuk National University School of Medicine, Chungbuk National University Hospital, Cheongju 28644, Korea; pedjhl@gmail.com; 13 Department of Pediatrics, Hanyang University Seoul Hospital, Seoul 04763, Korea; cord@hanyang.ac.kr; 14 Deparment of Pediatrics, Sungkyunkwan University School of Medicine, Kangbuk Samsung Hospital, Seoul 03181, Korea; hl.jung@samsung.com; 15 Department of Laboratory Medicine, Keimyung University School of Medicine, Keimyung University Dongsan Hospital, Daegu 42601, Korea; ksksmom@dsmc.or.kr; 16 Green Cross Genome, Yongin 16924, Korea; changski.md@gmail.com

**Keywords:** blood platelet disorders, high-throughput nucleotide sequencing, thrombasthenia, whole exome sequencing, whole genome sequencing

## Abstract

The diagnosis of inherited platelet function disorders (IPFDs) is challenging owing to the unavailability of essential testing methods, including light transmission aggregometry and flow cytometry, in several medical centers in Korea. This study, conducted by the Korean Pediatric Hematology Oncology Group from March 2017 to December 2020, aimed to identify the causative genetic variants of IPFDs in Korean patients using next-generation sequencing (NGS). Targeted exome sequencing, followed by whole-genome sequencing, was performed for diagnosing IPFDs. Of the 11 unrelated patients with suspected IPFDs enrolled in this study, 10 patients and 2 of their family members were diagnosed with Glanzmann thrombasthenia (GT). The variant c.1913+5G>T of *ITGB3* was the most common, followed by c.2333A>C (p.Gln778Pro) of *ITGB2B*. Known variants of GT, including c.917A>C (p.His306Pro) of *ITGB3* and c.2975del (p.Glu992Glyfs*), c.257T>C (p.Leu86Pro), and c.1750C>T (p.Arg584*) of *ITGA2B*, were identified. Four novel variants of GT, c.1451G>T (p.Gly484Val) and c.1595G>T (p.Cys532Phe) of *ITGB3* and c.1184G>T (p.Gly395Val) and c.2390del (p.Gly797Valfs*29) of *ITGA2B*, were revealed. The remaining patient was diagnosed with platelet type bleeding disorder 18 and harbored two novel *RASGRP2* variants, c.1479dup (p.Arg494Alafs*54) and c.813+1G>A. We demonstrated the successful application of NGS for the accurate and differential diagnosis of heterogeneous IPFDs.

## 1. Introduction

Inherited platelet function disorders (IPFDs) are a heterogeneous disease group associated with congenital defects in platelet function, including adhesion, activation, signal transduction, granule secretion, aggregation, and procoagulant activity [1,2]. Glanzmann thrombasthenia (GT) (OMIM #273800) is the most representative IPFD with a life-long bleeding phenotype [3]. It is an autosomal recessive disorder characterized by a failure of platelet aggregation due to quantitative or qualitative defects in the glycoprotein (GP)-IIb/GP-IIIa complex caused by mutations in *ITGA2B* or *ITGB3* [3]. There are several rarer types of IPFDs with similar phenotypes to GT, including Bernard–Soulier syndrome, acquired GT, leukocyte adhesion deficiency type III, and *RASGRP2*-related platelet dysfunction [4].

For the differential diagnosis of IPFDs, light transmission aggregometry (LTA) and flow cytometry (FC) are the first-line screening tests recommended by the International Society of Thrombosis and Haemostasis [5]. However, only very few hospitals in Korea can conduct LTA or FC due to the nature of the Korean national health insurance system. Thus, it is difficult to accurately diagnose and identify each IPFD case in Korea, and the prevalence of IPFDs in Korean patients and the distribution of their genetic abnormalities remain unknown. Thus far, only anecdotal cases of GT have been genetically confirmed and reported in Korea [2,6]. This study aimed to perform genetic confirmation and differential diagnosis of Korean IPFDs using next-generation sequencing (NGS).

## 2. Materials and Methods

### 2.1. Subjects and Data Collection

This multicenter observational, descriptive study was conducted by the Benign Hematology Committee of the Korean Pediatric Hematology Oncology Group (KPHOG) from March 2017 to December 2020. The experimental protocol was approved by the Institutional Review Board of Keimyung University Dongsan Hospital (Approval No. 2017-03-008), and this study was performed in accordance with the Declaration of Helsinki. Informed consent was obtained from all study participants before blood sampling.

We focused on non-syndromic IPFDs and included patients who met the following inclusion criteria: (1) platelet count in the normal range; (2) experienced primary hemostatic problems, such as petechiae, easy bruising, frequent or profound epistaxis, mucosal hemorrhage (including gum bleeding or menorrhagia), or bleeding that persists after vaccination, procedure, surgery, or childbirth; and (3) laboratory test results showing prolonged bleeding time, prolonged closing time in the Platelet Function Analyzer-100 (PFA^®^-100, Dade-Behring, Marburg, Germany), or platelet function defects by LTA. The exclusion criteria were as follows: (1) patients with bone marrow failure, congenital anomalies, albinism, immunodeficiencies, or other problems associated with syndromic inherited platelet disorders; and (2) those diagnosed with von Willebrand disease.

### 2.2. NGS

To identify pathogenic variants in IPFD and/or coagulation defect-related genes, targeted exome sequencing (TES) followed by whole genome sequencing (WGS) was performed. For TES, genomic DNA was extracted from EDTA whole blood. TruSight One (Illumina, San Diego, CA, USA) and Custom panel (Celemics, Seoul, Korea) were used for library preparation. Sequencing was done on the Illumina NextSeq500 platform (Illumina, San Diego, CA, USA). Alignment of sequence reads and variant calling using a pipeline were performed following the GATK Best Practices. Fastq trimming was done using Trimmomatic. Alignment was done using BWA-MEM (version 0.7.12), duplicated reads were marked using Picard (version 1.96, http://picard.sourceforge.net, accessed on 14 August 2014), local alignment, base quality recalibration, and variant calling were performed using the Genome Analysis Toolkit (GATK, version 3.5), and annotation was done using VEP88 (Variant Effect Predictor), dbNSFP v3.3.

For WGS, genomic DNA was extracted from EDTA whole blood and sequenced with paired-end reads on BGI-T7 platform. Libraries were constructed and sequenced with 100 bp paired-end reads on DNBSEQ-T7 (MGI Tech Co., Ltd., Shenzhen, China). The DNA sequence reads were trimmed using atropos (version 1.1.28) and aligned to reference sequence based on the public human genome build GRCh37/UCSC hg19. Reads alignment was done using BWA-MEM (version 0.7.17), duplicated reads were marked using biobambam2, base quality recalibration and variant calling were performed using HaplotypeCaller of Genome Analysis Toolkit (GATK, version 4.1.8), and annotation was done using VEP101 (Variant Effect Predictor), dbNSFP v4.1. The quality metrics of TES and WGS are shown in Table 1.

The variants were annotated with population (1000 Genomes Project [1000g], Exome Variant Server, Exome Aggregation Consortium [ExAC], Genome Aggregation Database [gnomAD], and Korean Reference Genome Database [KRGDB]) and disease databases (OMIM). For missense variants, in silico analysis was performed using SIFT, PolyPhen-2, and MutationTaster. Candidate variants, whose clinical significance has not yet been reported in the literature, were classified based on the guidelines for the interpretation of sequence variants from the American College of Medical Genetics and Genomics and the Association for Molecular Pathology (2015 ACMG/AMP classification) [7].

### 2.3. Genes of Primary Interest

Genes of primary interest included those associated with IPFDs and diseases with similar primary hemostatic problems, such as GT (*ITGA2B*, *ITGB3*), Bernard–Soulier syndrome (*GP1BA*, *GP1BB*, and *GP9*), adenosine diphosphate receptor P2Y_12_ defect (*P2RY12*), thromboxane A2 receptor defect (*TBXA2R*), Thromboxane synthase deficiency (*TBXAS1*), gray platelet syndrome (*NBEAL2*), Paris–Trusseau/Jacobsen syndrome (*FLI1*), Chediak-Higashi syndrome (*LYST*), Hermansky–Pudlak syndrome (*HPS1*, *AP3B1*, *HPS3*, *HPS4*, *HPS5*, *HPS6*, *DTNBP1*, *BLOC1S3*, and *BLOC1S6*), Scott syndrome (*ANO6)*, Quebec platelet disorder (*PLAU*), and other platelet type bleeding disorders (*P2RY12*, *TBXA2R*, *PTGS1*, *GFI1B*, *EPHB2*, *ACTN1*, *RASGRP2*, and *GP6*).

## 3. Results

Peripheral blood samples from 11 unrelated Korean patients (7:4 male:female ratio) with suspected IPFDs and their family members, as well as their bleeding symptoms and laboratory data, were collected during the study period. Using NGS, 10 cases were genetically confirmed as GT, whereas one was a case of platelet type bleeding disorder 18 (BDPLT18). The baseline clinical information of the IPFD patients is presented in Table 2, whereas laboratory test results and the identified genetic variants are summarized in Table 3. Most patients with IPFDs developed symptoms during infancy. Easy bruising, gum bleeding, whole body petechiae after birth, and persistent epistaxis were common symptoms.

Of the 11 Korean IPFD patients, 10 unrelated index patients and 2 of their family members were diagnosed with GT. The variant c.1913+5G>T of *ITGB3* was the most prevalent (9/20 variants, 45%); homozygotes were found in three unrelated subjects, and heterozygotes in other three subjects. The variant c.2333A>C (p.Gln778Pro) of *ITGB2B* was the second most common (3/20 variants, 15%) and was heterozygous in three unrelated subjects. In addition, known variants of GT, such as c.917A>C (p.His306Pro) of *ITGB3*, c.2975del (p.Glu992Glyfs*) of *ITGA2B*, c.257T>C (p.Leu86Pro) of *ITGA2B*, and c.1750C>T (p.Arg584*) of *ITGA2B*, were found. Four novel variants were discovered in four unrelated Korean GT patients: c.1451G>T (p.Gly484Val) and c.1595G>T (p.Cys532Phe) of *ITGB3* and c.1184G>T (p.Gly395Val) and c.2390del (p.Gly797Valfs*29) of *ITGA2B*. One patient was diagnosed with BDPLT18 (OMIM #615888) and harbored two novel variants of *RASGRP2*, namely c.1479dup (p.Arg494Alafs*54) and c.813+1G>A.

The four novel variants of GT are shown in Table 4. The NM_000212.2 (*ITGB3*): c.1451G>T (p.Gly484Val) variant found in family 1 was not detected in the population database and was predicted as deleterious by the in silico analysis. The same variant was also found in the patient’s younger sister, presenting the same hemorrhagic phenotypes of GT. Moreover, it was detected in *trans* with a known pathogenic variant (c.1913+5G>T of *ITGB3*), which was confirmed by Sanger sequencing in family 1. Thus, c.1451G>T of *ITGB3* was interpreted as likely pathogenic according to the 2015 ACMG/AMP classification [7]. Patient ID 1 and his sister presented obvious bleeding symptoms, such as severe epistaxis, easy bruising, and gum bleeding. Both siblings frequently visited the emergency room because of persistent epistaxis. At the time of visiting the hospital, they showed severe anemia and required red blood cell transfusions. Furthermore, both siblings had always presented petechiae and bruises all over their bodies, and their daycare teachers were suspicious about child abuse. Patient ID 1 had recurrent gum bleeding whenever he visited the dental clinic, thus we have prescribed prophylactic platelet transfusions before dental procedures.

The NM_000419.4 (*ITGA2B*): c.1184G>T (p.Gly395Val) variant found in family 8 was also not detected in the population database and predicted as deleterious by the in silico analysis. It was detected in *trans* with a known pathogenic variant (c.1750C>T [p.Arg584*] of *ITGA2B*), which was confirmed by Sanger sequencing in family 8. Thus, c.1184G>T of *ITGA2B* was interpreted as likely pathogenic according to the 2015 ACMG/AMP classification [7]. Patient ID 8 was enrolled in this study because of whole body petechiae developed after birth, which was accompanied by gum bleeding when she began teething. She also showed delayed wound healing and persistent bleeding when her finger was injured. She usually presented petechiae and bruises all over her body, so she switched to subcutaneous injections when vaccinated for bleeding prophylaxis.

The NM_000419.4 (*ITGA2B*): c.2390del (p.Gly797Valfs*29) variant has also not been reported in the population database. It existed in *trans* with a pathogenic variant (c.2333A>C [p.Gln778Pro] of *ITGA2B*) in family 9. Therefore, it was classified as pathogenic according to the 2015 ACMG/AMP classification [7], considering that this variant is a null variant with frameshift. Patient ID 9 was enrolled in this study because of obvious bleeding symptoms. She frequently bled from the gums after tooth brushing, was easily bruised, and repeatedly suffered from anal bleeding after defecation.

The last novel variant of GT, NM_000212.2 (*ITGB3*): c.1595G>T (p.Cys532Phe), was found in family 10 and was also absent in the control population. Previously, variants in which the amino acid at the same position was substituted as p.Cys532Tyr [8] and p.Cys532Arg [9] have been reported in patients diagnosed with GT. The variant c.1595G>T of *ITGB3* was predicted as deleterious by an in silico prediction tool and was detected in *trans* with a pathogenic variant (c.1913+5G>T of *ITGB3*), which was confirmed by Sanger sequencing in family 10. Therefore, it was classified as likely pathogenic according to the 2015 ACMG/AMP classification [7]. Patient ID 10 was enrolled in this study because of hematemesis associated with upper gastrointestinal bleeding.

## 4. Discussion

LTA and FC are among the first-line tests for the differential diagnosis of IPFDs. LTA is the gold standard method due to its high sensitivity and specificity [5]. However, it is expensive, time-consuming, and has complex operation with poor reproducibility, as it requires a large sample volume [5]. On the contrary, FC can be conducted with a limited sample volume and has high sensitivity and specificity [5]. However, it is expensive, laborious, and complex with high inter-laboratory variability [5]. GT is diagnosed using LTA based on the altered aggregation of epinephrine, ADP, and collagen, although the aggregation of platelets on ristocetin is normal, whereas FC is valuable for detecting antibodies to GPIIIa (CD61) or GPIIb (CD41) [10]. Unfortunately, these two important test methods are difficult to implement in every Korean medical center associated with the national health insurance system. In Korea, a system exists through which a private hospital performs necessary tests or treatments for patients, and then several months later, the private hospitals reimburse the cost from the national health insurance. Due to financial problems with Korean national health insurance, the reimbursements to the hospitals are very strict. In addition, the cost of reimbursements is set lower than the real costs for equipment or reagents required for the tests performed to diagnose IPFD in one person. Therefore, the burden of these costs has to be resolved by the private hospital itself, and thus becomes a deficit. Thus, we conducted NGS to accurately diagnose IPFDs in the KPHOG study.

Previously, Park et al. [6] found that among four unrelated Korean patients with GT, two were homozygous for c.1913+5G>T of *ITGB3* [6]. This variant has also been described as g.29107G>T by Tanaka et al. [11] and causes aberrant splicing, resulting in a premature stop codon [11]. It was repeatedly reported as a homozygote in two of four Korean GT patients; thus, it was proposed as a founder variant for Korean GT [6]. In this study, c.1913+5G>T of *ITGB3* was also the most common variant (6/10 GT patients) found as homozygote in 3 patients and heterozygote in 3 patients. Meanwhile, the variant c.2333A>C (p.Gln778Pro) of *ITGA2B* has a minor allele frequency (MAF) of 0.012% in East Asia according to the ExAC database and is classified as pathogenic in ClinVar. This variant has been reported not only in Korean patients [6] but also in Japanese patients [12]. Considering its relatively high MAF, we speculate that it is an Asian founder mutation of GT. In addition, the variant c.2975del (p.Glu992Glyfs*) of *ITGA2B* has been previously found in Korean patients with GT [6], whereas c.1750C>T (p.Arg584*) of *ITGA2B* is found in both Korean [6] and Japanese patients with GT [13]. Other known variants of GT among Japanese patients, including c.917A>C (p.His306Pro) of *ITGB3* [14,15] and c.257T>C (p.Leu86Pro) of *ITGA2B* [16], were also found in this study.

One IPFD patient was diagnosed with BDPLT18 based on the two novel variants of *RASGRP2* we identified. The NM_153819.1(*RASGRP2*): c.1479dup (p.Arg494Alafs*54) is a very rare variant with an MAF of 0.0098% in East Asia according to ExAC, whereas the NM_153819.1(*RASGRP2*): c.813+1G>A is also very rare and has not been reported in the population database. IPFD associated with *RASGRP2* mutation is a new category of disease called BDPLT18, a very rare disorder with severe primary hemostatic symptoms. There are approximately 20 *RASGRP2* mutations reported worldwide [17,18]. Pathogenic variants in *RASGRP2* lead to non-syndromic platelet dysfunction inherited in an autosomal recessive manner [19]. *RASGRP2* is located on chromosome 11q13.1 and encodes the Ras guanine-nucleotide releasing protein 2 (RasGRP2), also known as the calcium and diacyglycerol-regulated guanine exchange factor I [17]. RasGRP2 is an essential protein required for normal hemostasis that regulates the affinity between GPIIb-IIIa and fibrinogen via inside-out signaling in platelets [17]. IPFD associated with the truncation variant of *RASGRP2* shows a pattern of platelet aggregation defects similar to GT, prolonged PFA^®^-100 closure times, and reduced aggregation in response to ADP, epinephrine, and collagen, whereas aggregation with ristocetin is normal [20,21]. However, patients had normal expression of the major glycoprotein receptors [21].

This study has some limitations. First, not all laboratory tests for IPFD diagnosis could be performed in each hospital due to restrictions on services covered by the national insurance system. Second, although the novel variants found using NGS were defined according to the 2015 ACMG/AMP classification, further validation at the mRNA level was not performed. Based on the results of this study, KPHOG is planning to establish a Korean registry of inherited platelet disorders. In addition, KPHOG is seeking ways to expand the application of NGS for accurate diagnosis of IPFDs. Nevertheless, our findings are valuable because this is the first multicenter study of IPFDs using NGS in Korea.

## 5. Conclusions

In conclusion, we showed that the differential diagnosis of GT and other IPFDs is possible using NGS. Thus, applying NGS for heterogeneous IPFDs is useful for the accurate and differential diagnosis of IPFDs.

## Figures and Tables

**Table 1:** Quality metrics of TES and WGS for Korean patients with IPFDs.

Sequencing Method	ID	Quality of the Sequencing Reads	Sequencing Depth	Mapping Quality Threshold	Not Paired Read (%)	Missing Read (Unmapped) (%)
TES	1	31.9	83.7	30	0.28	0.16
TES	2	32.6	112.3	30	0.28	0.05
TES	3	33.4	145.5	30	0.57	0.06
TES	4	32.0	92.9	30	0.1	0
TES	5	33.0	84.5	30	0.32	0.06
TES	6	33.9	97.8	30	0.24	0.04
TES	7	32.3	79.6	30	0.32	0.08
TES	8	32.5	134.2	30	0.47	0.1
TES	9	33.5	106.0	30	0.36	0.06
TES	10	34.0	127.6	30	0.16	0.04
TES	11	32.1	94.7	30	0.29	0.16
Average		32.8	105.3	30	0.31	0.073
WGS	10	33.4	41.0	20	2.1	0.1

ID, identification; IPFD, inherited platelet function disorder; TES, targeted exome sequencing; WGS, whole genome sequencing.

**Table 2:** Baseline clinical information of Korean patients with IPFDs.

Clinical Characteristics	N
Male:female ratio	7:4
Age of symptom onset (months, range)	1 (0–48)
Bleeding symptoms	
Easy bruising	8
Gum bleeding	6
Whole body petechiae after birth	5
Persistent epistaxis	4
Delayed wound healing	2
Hematoma after vaccination	1
Bleeding after procedure	1
Melena	1
Anal bleeding	1
Hematemesis	1
Muscle hematoma	1

IPFD, inherited platelet functions disorder.

**Table 3:** Laboratory results and identified genetic variants in Korean patients with Glanzmann thrombasthenia and other inherited platelet function disorders.

ID	Plt (× 10^9^/L)	PFA-100, EPI (sec) (Reference)	PFA-100, ADP (sec) (Reference)	Light Transmission Aggregometry	Flow Cytometry	Gene	Genetic Variants	Classification
1	427	265 (60–180)	220 (50–110)	NA	Decreased CD41 expression	*ITGB3*	c.1913+5G>T	PV
							c.1451G>T (p.Gly484Val)	LPV
2	491	166 (60–180)	174 (50–110)	NA	NA	*ITGB3*	c.1913+5G>T [Hm]	PV
3	347	229 (60–180)	236 (50–110)	NA	NA	*ITGA2B*	c.2975del (p.Glu992Glyfs*)	LPV
							c.2333A>C (p.Gln778Pro)	PV
4	193	NA	NA	Decreased response to ADP, COL, EPI, normal response to RIS	NA	*ITGB3*	c.1913+5G>T [Hm]	PV
5	313	>300 (81–192)	157 (61–110)	NA	Complete deficiency of CD61/CD41a expression in platelet	*ITGB3*	c.917A>C (p.His306Pro)	PV
							c.1913+5G>T	PV
6	234	244 (82–182)	239 (62–109)	Decreased response to ADP, COL, EPI, normal response to RIS	NA	*ITGB3*	c.1913+5G>T [Hm]	PV
7	218	NA	NA	Decreased response to ADP, COL, EPI, normal response to RIS	Decreased CD41 expression	*ITGA2B*	c.257T>C (p.Leu86Pro)	LPV
							c.2333A>C (p.Gln778Pro)	LPV
8	392	223 (81–192)	240 (61–110)	NA	Decreased CD41 expression	*ITGA2B*	c.1750C> T (p.Arg584*)	PV
							c.1184G>T (p.Gly395Val)	LPV
9	309	234 (82–182)	212 (62–109)	Decreased response to ADP, COL, EPI, normal response to RIS	Decreased CD41 expression	*ITGA2B*	c.2390del (p.Gly797Valfs*29)	PV
							c.2333A>C (p.Gln778Pro)	PV
10	269	NA	NA	Decreased response to ADP, COL, EPI, normal response to RIS	Decreased CD41 expression	*ITGB3*	c.1913+5G>T	LPV
							c.1595G>T (p.Cys532Phe)	LPV
11	442	>300 (82–182)	>300 (62–109)	Decreased response to ADP, COL, EPI, normal response to RIS	NA	*RASGRP2*	c.1479dup (p.Arg494Alafs*54)	LPV
							c.813+1G>A	LPV

ADP, adenosine diphosphate; COL, collagen; EPI, epinephrine; Hm, homozygote; ID, idenfication; LPV, likely pathogenic variant; NA, not available; PFA, Platelet Function Analyzer; Plt, platelet; PV, pathogenic variant; RIS, ristocetin.

**Table 4:** Novel variants in Korean patients with GT.

ID	Gene	Genetic Variants	Frequency in Population Database	Prediction by the In Silico Analysis	Classification
1	*ITGB3*	c.1451G>T (p.Gly484Val) ^a,b^	0	Deleterious	LPV
8	*ITGA2B*	c.1184G>T (p.Gly395Val) ^a^	0	Deleterious	LPV
9	*ITGA2B*	c.2390del (p.Gly797Valfs*29) ^a^	0	Deleterious	PV
10	*ITGB3*	c.1595G>T (p.Cys532Phe) ^a,c^	0	Deleterious	LPV

GT, Glanzmann thrombasthenia; ID, identification; LPV, likely pathogenic variant; PV, pathogenic variant. ^a^ These variants were all detected in *trans* with known pathogenic variants of GT in the same family. ^b^ This variant was also found in the patient’s younger sister, presenting the same hemorrhagic phenotypes of GT. ^c^ Variants in which the amino acid at the same position was substituted and that were determined pathogenic in patients diagnosed with GT have been previously reported [8,9].

## Data Availability

Not applicable.
